# Barriers to self-care in women of reproductive age with HIV/AIDS in Iran: a qualitative study

**DOI:** 10.11604/pamj.2017.28.231.12385

**Published:** 2017-11-15

**Authors:** Fatemeh Oskouie, Farzaneh Kashefi, Forough Rafii, Mohammad Mehdi Gouya

**Affiliations:** 1 Nursing Care Research Center, School of Nursing and Midwifery,Iran; 2 Iran University of Medical Sciences, Tehran, Iran; 3 Iranian Centres for Communicable Disease Control (CDC); 4 Ministry of Health and Medical Education (MOHME), Iran

**Keywords:** Barriers, HIV, AIDS, Women, Self-care, Qualitative study

## Abstract

**Introduction:**

Although increasing attention is paid to HIV/AIDS, patients with HIV still experience several barriers to self-care. These barriers have been previously identified in small quantitative studies on women with HIV, but qualitative studies are required to clarify barriers to self-care.

**Methods:**

We conducted our study using the grounded theory methodological approach. A total of 28 women with HIV and their family members, were interviewed. The data were analyzed with the Corbin and Strauss method (1998).

**Results:**

The key barriers to self-care in women with HIV/AIDS included social stigma, addiction, psychological problems, medication side-effects and financial problems.

**Conclusion:**

Women with HIV/AIDS face several barriers to self-care. Therefore, when designing self-care models for these women, social and financial barriers should be identified. Mental health treatment should also be incorporated into such models and patients' access to health care services should be facilitated.

## Introduction

AIDS has led to a wide range of health, social and economic problems in human societies. Although more than 30 years have passed since the discovery of this disease, it remains a major challenge for health systems across the world and the prevention, treatment, care and control of AIDS remain a priority for health systems [1, 2]. In Iran, of all the HIV positive cases identified and reported from March to September 2015, 34% were women and 66% were men [3]. AIDS is a chronic disease that affects all aspects of a person's life. Infection with HIV during the reproductive stage of life affects the patient's own life and their family and their social life and imposes large costs on society [4]. According to the Global Health Observatory Data report, about $13 annually needs to be invested in treatment-related measures for every person who is HIV positive in low-to-middle income countries [5]. Women are estimated to account for 50% of HIV cases in the world [6]. Globally in 2015, 20% of new infections occurred in girls and women and 56% of these were in adult women [7]. In Iran, women who are HIV positive are often of poor socioeconomic status, poor health and have high levels of depression [8]. Young women with HIV have to deal with the challenges of the disease itself, and also often with pregnancy and its complications. They are also concerned with the risk of transferring the disease to their child during pregnancy and breastfeeding and with the child dying [9, 10]. Self-care is the ability of individuals, families and communities to promote health, prevent disease and maintain health and to cope with illness and disability with or without the support of a health-care provider [11]. Studies in women with HIV have shown associations between poor adherence to self-care principles and increased risk of opportunistic infections along with higher morbidity and mortality [12]. Many studies have examined the barriers to self-care that are caused by AIDS but have mostly been focused on the barriers to medication adherence and disease acceptance [13] or participation in support groups [14] and only one cohort study was found on the barriers to self-care [15]. Quantitative studies have paid little attention to the barriers to self-care and their consequences and the type of information documented in medical records and collected through questionnaires limited the findings of these studies. Therefore, a more qualitative approach is required to clarify all factors affecting barriers to self-care. Examining the context in which women who are HIV positive live is therefore crucial for better understanding the barriers to self-care. Any related intervention requires in-depth study of the cultural and social make-up of the country in question. Removing these barriers can improve access to healthcare facilities for these patients and increase their life expectancy appreciably. We aimed to identify barriers to self-care among women living with HIV/AIDS, using a grounded-theory approach.

## Methods

**Design and sampling**: Our study is part of a grounded theory study using the Strauss-Corbin approach (1998), which uses constant comparison, progressive coding and analysis and memoing. Grounded theory is a flexible method which allows for early findings instead of focusing on testing a predetermined hypothesis [16]. We began with purposive sampling and continued with snowball sampling from behavioral disease counselling centers affiliated to Iran, Tehran and Shahid Beheshti Universities of Medical Sciences. Notices about our research objectives were placed on notice boards in selected university-affiliated behavioral disease counseling centers, which provided all care procedures, including medication therapy, tests, physician visits and free counseling. We arranged with each center's physician to allow us to contact the patients on the phone or if they were members of the Positive Club, to speak to them in person and we explained the study objectives to them. Further arrangements were made for in-person interviews with the patients who were willing to take part in the study. The participants were 24 women of reproductive age, aged 20 to 46, with a definitive diagnosis of HIV/AIDS and no history of confirmed psychiatric disorder. They needed to understand the disease and be able to perform self-care. Before beginning the research, the participants signed and submitted informed consent forms. We mainly collected data through semi-structured in-depth interviews and also used field notes. All interviews were recorded in mp3 format with participants' prior consent. Data were collected over 10 months, between November 2016 and September 2017 and managed and organized in MAXQDA-10. Each interview began with general questions such as “Please tell us a little bit about your daily routines” and “How do you spend your days? With what activities?” The participants were then asked to define self-care, describe the barriers to self-care and discuss the actions they took in response to these barriers. We continued with more probing questions such as “Please elaborate on this point” and “What do you mean by that?” The interviews were conducted in a private room in the counseling centers and lasted from 35 to 65 min each (50 min on average). One or two interviews were held with each participant and by the end of the interview, the participant was given a $10 gift card as reimbursement for her time and transport.

**Data analysis**: We analyzed the data using the Corbin-Strauss approach (1998). Each interview was transcribed verbatim immediately after completion and its content analyzed. Data collection and analysis were performed concurrently using open, axial and selective coding [16]. In grounded theory, concept development starts with open coding, which involves line-by-line coding of the text. Using this technique, the text is broken down to its key elements. During analysis, constant comparisons are made to find relations between elements. Axial coding, simultaneously with data collection, is required to determine the main themes. In the process of data analysis, various techniques including theoretical sampling, theoretical memo writing, and constant comparative analysis are used to identify the existing categories [17]. We immediately recorded any question or new idea which arose during the open coding stage of analysis. These memos were helpful in selecting the next participant, editing the interview questions and analyzing the data. As the study progressed and the initial categories emerged, the data guided us to explore more about other people, such as the patient's spouse, mother and sister as caregivers. The interviews were considered complete when data saturation occurred.

**Trustworthiness**: To ensure the trustworthiness and validity of the data, Lincoln and Guba's evaluative criteria including credibility, transferability, dependability and confirmability were used [18]. We also used method triangulation and data source triangulation to check the validity of the data. The use of maximum variation sampling method increased the validity of our data. A member check was used by asking three participants from each recruiting center to read their own data and confirm its accuracy. We described the study's context as much as possible, so the readers were able to judge the transferability. To estimate stability criteria, the study process was audited by four experts who reviewed the study process and agreed on the findings.

**Ethics**: We observed ethics considerations by storing documents and transcripts on a secure computer with a pre-defined password that could only be accessed by the researcher and her supervisor. The voluntary nature of participation in the project, the confidentiality of the data and the anonymity of the participants were ensured throughout the study. The study was approved by the Ethics Committee of Iran University of Medical Sciences (105/5983).

## Results

Twenty-four women with HIV/AIDS and four caregivers, were interviewed in three counseling centers. The participants ranged in age from 20 to 46 years (mean, 33 years) and had differing marital status, methods of infection, education, economic status and durations of infection. This meant we achieved maximum diversity as well as theoretical saturation for our data [19] (Table 1). Data analysis revealed social stigma, addiction, psychological turmoil, medication side-effects and financial problems as key barriers to self-care.

**Table 1:** Demographic participants profile

Participant N°	Age	Educational level	Relationship status	Employment status	Time since HIV diagnosis	Risk factor for acquiring HIV
1	34	Less than high school	Married	Housekeeper	1 year	Injection
2	38	Less than high school	Married	Housekeeper	2.5 year	Husband
3	28	High school	Married	Housekeeper	1 year	Husband
4	30	More than high school	Divorce	Governmental job	1 year	Husband
5	46	Less than high school	Single	Private job	1 year	injection
6	28	High school	Separate	Private job	4 year	Husband
7	38	Less than high school	Divorce	Housekeeper	2 year	Husband
8	32	Less than high school	Divorce	Housekeeper	2 year	Injection & sex
9	33	Less than high school	Married	Housekeeper	3 year	Husband
10	34	More than high school	Single	Governmental job	8 year	Sex
11	37	More than high school	Divorce	Housekeeper	5 year	Husband
12	20	Less than high school	divorce	Housekeeper	3 year	Injection & sex
13	45	More than high school	Divorce	Governmental job	1 year	Sex
14	43	More than high school	Widow	Governmental job	4 year	Sex
15	42	Less than high school	Divorce	Housekeeper	1 year	Husband
16	28	More than high school	Divorce	Governmental job	2 year	Unknown
17	44	Less than high school	Married	Housekeeper	2 year	Husband
18	32	High school	Married	Housekeeper	3 year	Unknown
19	32	High school	Married	Housekeeper	1 year	Husband
20	37	Less than high school	Married	Private job	14 year	Husband
21	36	Less than high school	Married	Housekeeper	14 year	Husband
22	28	High school	Single	Housekeeper	1 year	Sex
23	39	Less than high school	Widow	Housekeeper	1 year	Injection
24	34	Less than high school	Single	Private job	4 year	Injection & sex

**Social stigma**: The social stigma of having AIDS was one of the key barriers to self-care, to the degree that these patients were hesitant about disclosure of their disease to their family, friends and colleagues, since they were uncertain about their reactions. Participant 6 said: I have been to the hospital on many occasions but never dared tell my family. Once, I went to Sevom-e Shaban Hospital but didn't tell my family, fearing they would find out. Now I have a lump of fat in my back that needs an operation and my biggest problem is that I can't tell anyone to have them come and take care of me in the hospital. My doctor suggested that I tell my surgeon, which I did and he accepted it with great ease and made an appointment for my surgery. But I can't tell my family, cause, say, if I ask my sister to go with me, she is very likely to find out once she's in the ward with me and she may not react well. So I have to go with my husband. Participant 12 said: I prefer to self-medicate rather than go anywhere and have to disclose my illness. Every time I come here, I try to come when it's not crowded. Before, when the center was inside, it used to get very crowded and I didn´t like that. The way people think is that, every time someone passes by you, you are burdened, because they know why you are here.

**Addiction**: Addiction was another barrier to self-care in these patients. Our results that drug addicts have a chaotic life and multiple unprotected sexual relationships. Some of them had been imprisoned several times for addiction. Participant 1, an ex-addict who has now successfully quit reported: it was first my husband who got addicted to opium. Then I also started smoking opium with him and moved on to crack and finally to crystal meth. We never had any tests done during this period and so we didn't know that we had HIV. It hurts badly when I'm reminded of that. Participant 13, an ex-addict who is supported by the social welfare organization described her experience: when I married my husband, he got injections, but I didn't, I only smoked crystal meth. It was my husband who got me addicted. He said “smoke, it'll make you feel better”. My family are also addicts. My father used crack till the day he died. My mother is also an addict; and so is my married sister. My other sister and brother are now under the social welfare. I have no news of my mother. I was brought up on the streets since primary school. We slept on the streets and knew nothing about this disease.

**Psychological turmoil**: Most participants had experienced shock, denial and anger when first diagnosed with HIV and it had led to psychological problems for some, which we identified through depression, anxiety and feelings of despair and disappointment.

**Depression**: Depression was a key psychological barrier to self-care in HIV-positive patients, as some lacked a promising image of the future. They were unable to live in peace due to anxiety and more importantly, the associated stigma. Participants who had depression stated that they mostly slept through the day and had no interest in visiting a counseling center, Positive Club or following up on their own condition. Participant 2 explained: at first, I wanted nothing from life and just wanted to die sooner. I wanted to have nothing to do with my husband, because I used to think he was my enemy for giving me the disease. I did the house chores just because I had to. Then I tried to come to terms with how this is a disease nobody can do anything about. They say cancer is horrible; this is worse.

**Anxiety**: Anxiety was another psychological barrier to HIV self-care, imposing great pressure on the women. Although the severity varied, most participants suffered some degree of anxiety due to uncertainty about the future and concern about their children's prospects. Participant 4 said: sometimes I feel my life is over and I wonder what will happen to Gandom (her daughter). I don't want her to be brought up by somebody else.

**Despair and disappointment**: Feelings of despair and disappointment were also barriers to self-care. Most patients had lost hope at different stages and would distance themselves from society when they were alone at home or when they had no effective interpersonal relationships. Negative thoughts and lack of hope for a cure were among the issues that exacerbated these feelings. Participant 2 described her experience: this disease gives you a certain feeling. As if you're always waiting for it (to end). Because you can talk to no one about it and it has no cures, so you worry about the virus growing in you and you just have to wait to die. That bad of a feeling.

**Medication side-effects**: Medication side-effects were also barriers to self-care. Some participants complained about side-effects such as hair loss, nausea and aggressiveness. Participant 7 said: I experienced such severe hair loss when I first began taking the meds that I had to wear a wig to parties. Participant 20 explained: I sometimes stop taking my medications, because of their side-effects; they make me aggressive. I treat my child so badly for saying anything at all. That's why I stop taking them, it's for my kid's sake. My double-dose pills give me diarrhea. I've asked for a single dose instead, but they say it's impossible because I have drug resistance and so should carry on with the double dose. Some other participants were not happy about their multiple-drug regimen and considered taking pills a burden and said they avoided taking medications in any way they could.

**Financial problems**: Although all the HIV tests and medications were free of charge at the behavioral disease counseling centers in Iran, financial problems were another barrier to self-care in the participants. HIV-positive women have low incomes and most are not able to continue their jobs because of fatigue and depression. Some had lost their jobs when their employer learned of their disease. Others had had to undergo complementary diagnostic tests due to their low CD4 count and high viral load despite their treatments, which burdened the patients with even greater expenses. Participant 7 described her experience: treatment costs are very high for this disease. Right now, my son and I have to undergo drug resistance and viral load tests (our CD4 has not changed), which will cost us each one million Tomans. The tests are just too expensive. Participant 13 was another patient who experienced financial distress. She worked late and on holidays to make enough money to support her two children after her divorce and therefore she lacked adequate sleep and nutrition and had neglected herself. We found financial problems to be the most important barrier to self-care, along with the social stigma associated with HIV. Nevertheless, some women were serious about their self-care and disease follow-up despite their numerous problems and the will to look after their children was their main motivation.

## Discussion

Adherence to treatment and self-care is a key factor in successful control of HIV/AIDS. We conducted our study to explain the barriers to self-care in women of reproductive age with HIV/AIDS. Our results showed that social stigma, addiction, psychological turmoil, medication side-effects and financial problems were the key barriers to self-care.

**Social stigma**: One of the main barriers to self-care and a key concern in these patients, was the social stigma of AIDS, which restricted their social presence. Stigma remains one of the biggest problems in relation to HIV/AIDS throughout the world [14]. Despite the benefits of self-care for chronically ill patients, they rarely visit counseling centers because they fear having their illness revealed to others and some are indifferent to their own health [14]. Other studies suggest that some patients do not keep medical appointments because they do not want to disclose their disease. The stigma associated with AIDS affects disclosure of HIV, access to social support and feelings of health and well-being [13, 20]. Removing this barrier of stigma and raising awareness in society about the disease and its pathogenesis and transmission is necessary.

**Drug addiction**: another barrier to HIV/AIDS self-care was drug abuse and addiction. Most participants who were drug addicted had no support because their family members were also drug addicts. Addiction had also exposed these patients to high-risk sexual behavior and unprotected sex. Many had death ideation, and the life-threatening nature of the disease meant that participant 3, who abused drugs, had increased her drug use when first diagnosed with HIV, which had worsened her condition. Previous studies have shown that HIV-positive addicts experience high levels of anxiety due to their own destructive behaviors and the vulnerability caused by addiction [21]. People with high levels of anxiety cannot take proper care of themselves. Most women in our study had successfully quit their drug habit. Previous studies have also shown that anxiety can exacerbate drug abuse in these patients and accelerate their death [21].

**Psychological turmoil**: Another barrier to self-care in these patients was psychological turmoil, which meant the participants led chaotic lives. Previous studies suggest that, in addition to physical consequences, chronic diseases are associated with psychological harms [22]. Patients who feel sicker or are depressed care less about their self-care and treatment [23]. In chronically ill patients, depression is common and life expectancy is reduced [24]. In Iran, the presence of psychiatrists and counselors in counseling centers contributes significantly to improvement of disease outcomes.

**Medication side-effects**: Medication side-effects were another barrier to self-care, according to participants. Multiple-drug regimens led to side-effects in some patients that, although temporary, led to treatment discontinuation. One study reported that a cause of treatment non-compliance in HIV/AIDS patients was due to physical changes caused by the medications and fear of disclosure of their disease [21].

**Financial barriers**: Financial problems, including the costs of commuting to counseling centers, vitamin supplements and proper nutrition were another barrier to self-care in these patients. Some participants were unable to continue their jobs outside the house because of fatigue. This exposed them to financial problems and participants who headed their households were faced with great challenges in supporting themselves and their children. According to previous studies, adherence to treatment and self-care is better in centers that provide free support services such as transport and patient monitoring [20, 23]. In our study, patients who were concerned about their finances had neglected their self-care. Studies have shown that financial pressure caused by chronic diseases are often overlooked [25] and may cause patients to discontinue treatment [26] and prevent them enjoying life [27]. In our study, most participants were unable to travel for leisure and many had to work long hours. Women who were single mothers paid less attention to themselves and their health was therefore at risk [28]. Female gender roles such as child care and housework cause women to neglect self-care; this is especially so for chronically ill patients [29].

**Limitations**: Our research was the first of its kind in Iran that investigated the magnitude of barriers to self-care. We used in-depth interviews that allowed us explore the participants' feelings and perspectives on barriers to self-care. The strengths of this study were detailed description of the context, maximum variation and theoretical sampling. However, since the findings were extracted from other qualitative research designs, they cannot be generalized. The limitation of this study was selecting the participants from HIV clinics. As a result, the perceptions of women with lower level of participation in their communities might have been neglected.

## Conclusion

Understanding the barriers to self-care among women with HIV/AIDS can help care teams, especially midwives, in providing better care to these women. Our findings provide health policy-makers with useful information about how to overcome the barriers to self-care in women with HIV/AIDS.

### What is known about this topic

- Prevalence of HIV among women in Iran is alarmingly high;
- There are common behaviours for acquisition and transmission of HIV among women;
- Many women with HIV experience inadequate self-care.

### What this study adds

- Addiction is a barrier to self-care and adherence to treatment and follow-up among women with HIV;
- Psychological problems can cause isolated in women with HIV;
- Financial problems and social stigma are two other barriers to self-care in these women.

## Competing interests

The authors declare no competing interests.
